# Effects of oil quality and antioxidant supplementation on sow performance, milk composition and oxidative status in serum and placenta

**DOI:** 10.1186/s12944-017-0494-6

**Published:** 2017-06-07

**Authors:** Guoqi Su, Junmei Zhao, Guangbo Luo, Yue Xuan, Zhengfeng Fang, Yan Lin, Shengyu Xu, De Wu, Jun He, Lianqiang Che

**Affiliations:** 1 Institute of Animal Nutrition, Sichuan Agricultural University, No. 46 Xinkang Road, Yaan, Sichuan 625014 China; 2 Novus International, Inc. 20 Research Park Drive, St. Charles, MO 63304 USA

**Keywords:** Sows, Oxidative stress, Antioxidant, Placenta

## Abstract

**Background:**

The aim of this study was to determine the effects of oil quality and antioxidant (AOX) supplementation on sow performance, milk composition and oxidative status.

**Methods:**

A total of 80 PIC (PIC breeding, 3 ~ 5 parities) sows with similar body condition were allocated to four groups (*n* = 20), receiving diets including fresh corn oil, oxidized corn oil, fresh corn oil plus AOX and oxidized corn oil plus AOX, respectively, from d 85 of gestation to d 21 of lactation. AOX was provided at 200 mg/kg diet and mixed with corn oil prior to dietary formulation.

**Results:**

The results showed that sows fed oxidized corn oil had significantly lower feed intake (*P* < 0.05) during lactation period. Feeding oxidized corn oil markedly decreased (*P* < 0.05) the contents of protein and fat in colostrums and milk, but the addition of AOX in oxidized corn oil prevented the decrease on protein content of colostrums. Moreover, sows fed oxidized corn oil had significantly lower serum activities of total SOD and Mn-SOD across lactation (*P* < 0.05). In contrast, addition of AOX to oxidized corn oil tended to inhibit the production of MDA (*P* = 0.08) in sows across lactation relative to fresh oil. Intriguingly, the placental oxidative status was affected by oil quality and AOX supplementation, as indicated by the markedly increased placental gene expression of GPX and SOD (*P* < 0.05) in sows fed oxidized corn oil but normalized by supplementation of AOX.

**Conclusion:**

In conclusion, feeding oxidized corn oil did not markedly affect reproductive performance in addition to decreasing feed intake during lactation. Milk composition and systemic oxidative status were deteriorated in sows fed oxidized corn oil and partially improved by AOX supplementation. Moreover, placental antioxidant system of sows may have an adaptive response to oxidative stress, but normalized by AOX.

## Background

The balance between beneficial and harmful effects of free radicals is important for animal health, which is achieved by controlling the redox status in vivo [1, 2]. However, overproduction of free radicals may exceed the capability of antioxidant system, resulting in oxidative stress which can damage cellular lipids, proteins or DNA, thus inhibiting their normal function [3]. Growth performance, liver and intestinal functions of animals were impaired under oxidative stress [4–7].

Dietary oxidized fat has been recognized as an important factor to induce oxidative stress and decrease performance in food animals such as pigs [8], poultry [9, 10] and aquaculture [11], however, very limited data was reported in dam animals. A recent study showed that sows suffered systematic oxidative stress due to the lower antioxidant nutrients and total antioxidant capability during late gestation and lactation [12], when there was increased transfer of antioxidant nutrients to fetus [13]. Furthermore, in order to increase energy density for fetal growth, vegetable oil has been widely used as an energy source in late gestation and lactation diets, which however could be oxidized to produce ROS and induce oxidative stress [14]. Therefore, sophisticated antioxidant system is required to stabilize ROS level. Synthesized antioxidant is being used to control ROS by scavenging free radical and preventing the accumulation of free radicals in the body [15]. Previous studies have indicated dietary antioxidant supplementation prevented excessive production of ROS and improved performance as well as antioxidant status in cow lactation [16] and broilers [17].

Therefore, the objective of this study was to determine the effects of feeding oxidized corn oil with or without antioxidant supplementation on sow performance, milk composition and oxidative status of sows in late gestation and lactation.

## Materials and methods

### Animals and experimental design

This study was conducted at Gaojing animal experiment base, and followed the Guide for the Use of Animals for Experiment issued by Ministry of Science, China.

A total of 80 sows (PIC breeding, 3 ~ 5 parities) with similar body condition (backfat thickness at 18 ± 0.3, Mean ± SE) were allocated to four groups (*n* = 20), receiving corn-soybean meal diet mixed with corn oil at 2% inclusion from d 85 of gestation until d 21 of lactation. Experiment was carried-out as a factorial arrangement (2 × 2), by 2 types of corn oil (fresh or oxidized corn oil) with or without antioxidant (AOX), respectively. AOX (AGRADO® Plus, to provide Ethoxyquin, Citric acid and Tertiary butyl hydroquinone) was supplied by NOVUS International Inc. (St. Charles, MO, USA). The four experimental treatments were defined as follows: Treatment 1) Basal diet containing fresh corn oil (FO); Treatment 2) Basal diet mixed with oxidized corn oil (OO); Treatment 3) FO plus 200 mg/kg AOX; Treatment 4) OO plus 200 mg/kg AOX.

### Experimental diets

By referring the methods from Fernandez-Duenas [8], oxidation of corn oil was achieved by continuously bubbling air at a rate of 80 L/min (liters per minute) and heating at 95 C° for 72 h. Peroxide values were determined as described by National Analytical Criteria (GB-T 5009.37–2003), China. Corn oil was oxidized to a target peroxide value of 630 mEq/kg of oil, thereafter oxidized oil was diluted with fresh oil to reach a target peroxide value of 250 mEq/kg oil or a final feed peroxide value of 5 mEq/kg feed. Both control and treatment oils were stored under refrigeration (4C°) to prevent further oxidation.

Experimental diets were produced weekly. Prior to production, AOX was added in fresh or oxidized corn oil. Thereafter, AOX protected or unprotected corn oil was mixed with the basal diet for the final experimental diets. Basal diet was formulated to meet the nutrient requirements of sows according to NRC (1998) (Table 1). Nutrient values of ingredients were referred to <<Nutrient Values of Chinese Feed Ingredients>> (18th edition, 2008).

**Table 1:** Composition and nutrient levels of diet (air dry basis, %)

Ingredients, %		Nutrient levels	
Corn, 7.8% CP	65.50	Crude protein,%	17.00
Soybean meal,43% CP	22.00	DE, Mcal/kg	3.30
Wheat bran	4.50	DM,%	87.67
Corn oil	2.00	EE,%	5.00
Fish meal,65% CP	2.00	CF,%	2.60
CaHPO_3_	1.20	Ash,%	5.00
CaCO_3_	0.90	Ca,%	0.82
Choline,50%	0.10	Total P,%	0.60
NaCl	0.30	Available P,%	0.40
NaCHO_3_	0.20	Lys,%	1.15
L-Lys,98%	0.37	SAA,%	0.63
Val,98%	0.15	Met,%	0.34
Thr,98%	0.11	Thr,%	0.74
MHA® ^a^	0.07	Trp,%	0.23
Trp,98%	0.04		
Premix ^b^	0.50		
Total	100.00		

^a^MHA® (Methionine Hydroxy Analogue, Novus International Inc., USA) was taken as 84% methionine activity

^b^Trace minerals and vitamins premix provided per kilogram of diet: Zn 100 mg; Cu 30 mg; Fe 100 mg; Mn 40 mg; I 0.15 mg; Se 0.15 mg; Vitamin A 2000 IU, Vitamin D_3_ 800 IU, Vitamin E 40 mg, Vitamin B_1_ 3 mg, Vitamin B_2_ 10 mg, Vitamin B_6_ 4 mg, Vitamin B_12_ 40 μg, Nicotinic acid 50 mg, Pantothenic acid 30 mg, Folic acid 4 mg, Biotin 0.45 mg, Choline chloride 750 mg

### Management

All pregnant sows were housed individually in stalls (2.5 × 1.6 m) until day 3 before farrowing when sows were transferred to farrowing unit (2.2 × 1.8 m). The experimental diets (from day 85 of gestation to day 21 of lactation) were supplied twice a day (08:00 and 17:00) at 3.5 kg/day until day 3 before farrowing, then feed allowance was reduced by 0.5 kg/day until farrowing day when no diet was provided. During lactation, diets were supplied three times a day (08:00, 11:30 and 17:30) and was started at 2.0 kg, then increased by 0.5 kg/day during the first week as previously reported [18]. Afterwards, sows had free access to diets. Water was sufficiently supplied during gestation and lactation. During 24 h post-farrowing, litter size was equalized within treatment to achieve 10 ~ 11 pigs per sow, all pigs had free access to creep feed from day 7 after birth and weaned at 21 day of age. Room temperature of gestation and farrowing units were controlled at approximately 22 °C and 26 °C, respectively.

### Measurements

#### Sow backfat thickness

Sow backfat thickness was measured at 60 mm left side of the dorsal mid line at the last rib level (P2) using ultrasound (Piglog 105, SFK-Technology, Herlev, Denmark) and recorded at day 85 of gestation, d 1 and 21 of lactation.

### Sow performance

Litter performance (total pigs born, pigs born alive, litter weight at parturition and day 21 of lactation) were recorded. The supplied and residual feed in pens every day were recorded to calculate the daily feed intake of sows during lactation. Placental efficiency (ratio of newborn weight to placental weight) was calculated at farrowing.

### Oxidative status

Systemic oxidative stress was determined by measurement of antioxidant nutrients such as vitamin E and vitamin C, enzyme activities such as glutathione peroxidase (GPX), superoxide dismutases (SOD) including total, CuZn- and Mn-SOD. Malondialdehyde (MDA), as the product of oxidative damage to lipids, was also estimated. All indexes were based on serum samples (*n* = 8) collected from ear vein at different gestational age (d 85 of gestation, d 1 and 21 d of lactation). Colostrum, milk from d 7 and 21 of lactation (*n* = 8) were collected for analysis of milk composition (Automatic Milk Composition Analyzer, Zhejiang, China). The antioxidant parameters were analyzed by assay kits from Nanjing Jiancheng Institute of Bioengineering (Nanjing, Jiangsu, China), following the instructions of kits. All samples were measured in triplicate. After thawing serum samples in ice-cold buffers, VE, VC, activities of antioxidant enzymes and MDA level were determined using colorimetric methods with spectrophotometer.

### Gene expression

The placental oxidative status is assessed by mRNA level of the antioxidant enzymes (GPX, SOD, CAT and NOS) in placental tissues collected at farrowing (*n* = 8). After farrowing, placenta tissues (2 cm × 2 cm) by about 5 cm far away from joint site of umbilical cord to placenta were collected and snap frozen in liquid nitrogen. Total RNA was extracted from frozen placenta tissue using Trizol Reagent (Invitrogen, Carlsbad, CA, USA) according to the manufacturer’s instructions. Quality and purity of RNA samples were assessed by electrophoresis on 1.0% agarose gel and nucleic acid analyzer (Beckman DU-800, Los Angeles, CA, USA) as previously reported [19]. RT-PCR was performed in triplicate to amplify the target gene and the reference gene (β-actin and GAPDH) using one step SYBR Prime Script TM RT-PCR kit II (Catalog no. DRR086A, Takara, Japan) using reverse transcription-polymerase chain reaction (ABI 7900HT, Applied Biosystems). The sequences of primers and length of the products are shown in Table 2. The reaction mixture (10.0 μL) contained 5.6 μL of freshly premixed one step SYBR Green RT-PCR Master mix and Prime Script TM Enzyme Mix, 0.8 μL of the primers and 150 ng of RNA template. The RT-PCR program was designed with 1 cycle of 42 °C for 5 min, 1 cycle of 95 °C for 10 s, and 40 cycles of 95 °C for 5 s and 60 °C for 34 s, followed by the dissociation step at 95 °C for 15 s, 60 °C for 60 s, and 95 °C for 15 s. At the end of amplification, melting curve analysis was performed to identify amplification specificity. For each of the target genes, the CT values of all the samples were then calculated by subtracting the average CT of the corresponding normal body weight group or normal body weight group with formula feed. The CT values were converted to fold differences by raising 2 to the power – CT (2^- CT^) according to Livak K J, et al. [20].

**Table 2:** Primer sequences of target and reference genes

Gene	Primer sequence(5′-3′)	Product (bp)	Genebank accession
GP_X_	F: GCTCGGTGTATGCCTTCTCT	103	NM_214201.1
	R: AGCGACGCTACGTTCTCAAT		
NOS	F: AGAGCCTCTGGACCTCAACA	136	NM_001143690.1
	R: CTCACAGCAGAGTTCCACCA		
SOD	F: GAGACCTGGGCAATGTGACT	189	GU944822.1
	R: CTGCCCAAGTCATCTGGTTT		
CAT	F: ACTTCTGGAGCCTACGTCCT	93	NM_214301.2
	R: ATCCGTTCATGTGCCTGTGT		
β-actin	F: GGCGCCCAGCACGAT	66	DQ845171.1
	R: CCGATCCACACGGAGTACTTG		

*GPx* glutathione peroxidase, *NOS* nitric oxide synthase, *SOD* superoxide dismutase, *CAT* catalase

### Statistics

Data were analyzed using the MIXED procedure of SAS (SAS Inst. Inc., Cary, NC). For sow performance, the model included main effects of Oil (FO vs. OO) and AOX (with or without AOX) as well as their interactions. For parameters on milk composition and serum oxidative status which were taken over time, repeated measure data were analyzed using the mixed procedure with sow within treatment as the subject and the error term to test for main effects and interaction, the residual error was used to test for day and day by treatment interaction. All means are least squares means. *P* < 0.05 was considered statistically significant.

## Results and discussion

In this study, we formulated sow diets with oxidized corn oil, containing 5 mEq/kg peroxide value, which is relatively lower than the value of 9 mEq/kg diet used in growing pigs by Fernandez-Duenas [8]. However, sows have 2 ~ 4 times higher feed intake than that of growing pigs, and an increased systemic oxidative stress and reduced concentration of antioxidant nutrients had been observed during late gestation and lactation of sows [21, 22]. In this study, therefore, we are the first time to determine the effects of oxidized oil with this peroxide value on the litter performance and oxidative status of sows during late gestation and lactation.

As shown in Table 3, feeding oxidized oil did not markedly affect litter performance and placental efficiency at farrowing. Consistently, the number of total pups and pups born alive were not markedly affected in rats receiving oxidized oil [23]. However, feeding oxidized oil significantly decreased daily feed intake of lactating sows (*P* = 0.04). Previous reports showed that diet with inclusion of oxidized choice white grease resulted in a negative effect on feed intake, affecting weight gain of nursery pigs [24]. Likewise, feed intake was markedly decreased in broilers receiving oxidized oil [25], the decreasing feed intake could be ascribed to the rancid aroma developed by oxidized fat or oils [24, 26, 27].

**Table 3:** Effects of oxidized oil with or without antioxidant (AOX) on sow performance

	Oil quality		AOX		SEM	*P* value		
	FO	OO	FO + AOX	OO + AOX		AOX	Oil	AOX × Oil
Total litter size	12.72	12.63	12.69	12.83	0.42	0.84	0.94	0.78
Live litter size	11.00	10.95	10.38	10.91	0.39	0.34	0.51	0.42
Mommified	0.67	0.52	0.63	0.46	0.22	0.78	0.5	0.95
Dead	1.06	1.16	1.69	1.46	0.23	0.12	0.78	0.49
Litter weight	15.43	15.01	14.97	15.31	0.50	0.93	0.95	0.45
Placental efficiency	4.72	4.84	4.85	4.81	0.28	0.87	0.91	0.80
Average Birth weight	1.42	1.41	1.47	1.43	0.06	0.64	0.72	0.90
Initial pigs, No.	10.56	10.42	10.5	10.58	0.11	0.59	0.84	0.36
Initial litter weight, kg	15.35	14.52	15.53	15.39	0.50	0.29	0.34	0.49
Initial body weight, kg	1.46	1.39	1.47	1.45	0.05	0.40	0.33	0.68
Weaning pigs, No.	9.33	9.6	9.56	9.57	0.25	0.70	0.58	0.62
Litter weight at weaning, kg	53.62	51.86	54.55	53.79	2.30	0.54	0.59	0.34
Weaning body weight, kg	5.74	5.39	5.68	5.62	0.15	0.60	0.21	0.83
Body weight gain, kg	4.28	3.94	4.29	4.15	0.15	0.46	0.13	0.51
Daily feed intake of sows, kg	4.89	4.48	4.86	4.72	0.13	0.40	0.04	0.30
Backfat thickness at d 85	17.4	18.2	17.8	18.8	0.89	0.69	0.47	0.20
Backfat thickness at farrowing	18.6	19.8	19.1	19.8	0.70	0.64	0.70	0.15
Backfat thickness at d 21	16.3	16.9	16.4	16.8	0.85	0.53	1.00	0.35
Backfat lost	2.3	3.3	2.7	3.0	0.60	0.92	0.28	0.54

Treatments: FO means fresh oil; OO means oxidized oil; FO + AOX means fresh oil plus AOX; OO + AOX means oxidized oil plus AOX. *n* = 20

Considering sow feed intake would affect milk production and composition, we further measured the milk composition. As indicated in Table 4, compared with sows receiving fresh corn oil plus AOX (FO + AOX), feeding oxidized oil markedly decreased the content of protein in colostrums (−8%, *P* < 0.05), but this did not appear in sows receiving oxidized oil plus AOX, suggesting the potential capability of AOX on improving colostrum quality. Moreover, sows receiving oxidized oil had markedly lower (*P* = 0.01) milk fat content across the 21 d of lactation, but again this difference did not appear in sows receiving oxidized oil plus AOX. Similarly, lactose content at d 21 of lactation was lower (*P* = 0.1) in sows fed oxidized oil, but no significant difference was observed after AOX supplementation. These findings strongly indicate the impairment of oxidized oil on milk composition that can be alleviated by dietary supplementation of AOX,which was similar with previous report [28] . The deteriorated milk composition by feeding oxidized oil could be associated with the lower metabolic substrates for synthesis of milk in mammary gland [23]. In contrast to the increased milk fat yields in cows receiving oxidized oil [16], in this study, milk fat was significantly decreased by feeding oxidized oil. On one hand, it may be ascribed to the lower capability of digestion and retention of fat when it is oxidized [29, 30], on the other hand, milk triacylglycerol synthesis depends on the availability of fatty acids in mammary gland, but feeding oxidized fat decreased the concentration of triacylglycerols in the milk by lowering uptake of fatty acids from triacylglycerol rich-lipoproteins and non essential fatty acids into the mammary gland [23, 31].

**Table 4:** Effects of oxidized oil with or without antioxidant (AOX) on sow colostrum and milk composition

	Oil quality		AOX		SEM	*P* value						
	FO	OO	FO + AOX	OO + AOX		AOX	Oil	Day	AOX × Oil	Day × AOX	Day × Oil	Day × AOX × Oil
Protein												
Overall Mean	5.31	5.14	5.33	5.20	0.10	0.52	0.18	0.00	0.91	0.95	0.08	0.38
Day 1 of lactation	7.47^ab^	7.09 ^b^	7.72^a^	7.23^ab^	0.17							
Day 7 of lactation	4.09	4.14	4.09	4.19	0.17							
Day 21 of lactation	4.23	4.06	4.20	4.31	0.17							
Fat												
Overall Mean	6.34	5.80	6.86	5.82	0.10	0.37	0.01	0.00	0.41	0.31	0.98	0.13
Day 1 of lactation	5.56	4.38	5.31	4.99	0.48							
Day 7 of lactation	6.25^b^	6.39^b^	8.02^a^	6.27^b^	0.48							
Day 21 of lactation	7.32	6.63	7.26	6.23	0.48							
Lactose												
Overall Mean	8.09	8.06	8.02	8.22	0.17	0.85	0.63	0.00	0.73	0.7	0.08	0.96
Day 1 of lactation	10.58	10.97	10.38	10.97	0.29							
Day 7 of lactation	6.61	6.80	6.57	6.78	0.29							
Day 21 of lactation	6.96	6.44	7.11	6.76	0.29							
Non-fat solid												
Overall Mean	14.15	14.08	14.03	14.57	0.2	0.52	0.39	0.00	0.49	0.78	0.05	0.95
Day 1 of lactation	19.59	20.3	19.73	20.97	0.43							
Day 7 of lactation	10.95	11.09	10.94	11.24	0.43							
Day 21 of lactation	11.6	10.88	11.44	11.06	0.43							

Means in rows with different superscripts markedly differ, *P* < 0.05. *n* = 8

Treatments: FO means fresh oil; OO means oxidized oil; FO + AOX means fresh oil plus AOX; OO + AOX means oxidized oil plus AOX

^a,b^Mean values within a row with unlike superscript letters were significantly different (*P* < 0.05)

In order to determine dietary oxidized oil on oxidative status of sows, we further analyzed systematic and placental antioxidant parameters. As indicated in Table 5, antioxidant nutrients (VE and VC) did not markedly differ among groups but significantly depended on the period of gestation, suggesting the transfer of antioxidant nutrients to fetus for their rapid growth in late gestation [13]. This finding is supported by the results of Berchierironchi et al., who found systematic antioxidant nutrients were substantially reduced at d 110 of gestation [21]. Moreover, sows receiving oxidized oil had markedly lower activities of total SOD and Mn-SOD over time (*P* < 0.05), particularly decreased SOD activity at d 1 of lactation compared with sows receiving fresh oil and fresh oil plus AOX (*P* = 0.01 and 0.07, respectively). In contrast, dietary AOX supplementation to oxidized oil normalized activity of total SOD, and tended to increase GPx activity (+19%, *P* = 0.12) compared with oxidized oil at d 21 of lactation. Taken together, we postulated that the peroxides from oxidized oil may exhaust endogenous antioxidant enzymes and produce excessive end products of oxidation. MDA, as an indicator of lipid peroxidation, was indeed higher in sows fed oxidized oil across lactation period (*P* = 0.06). Likewise, supplementation of AOX to oxidized oil inhibited the production of MDA, as evidenced by the lower MDA level (*P* = 0.08) in sows receiving AOX relative to sows without receiving AOX across lactation. Therefore, the improvement of AOX on oxidative status may be ascribed to its reaction to reactive oxygen molecules and end products of oxidation, preventing insult of peroxides to sows and sparing endogenous antioxidant defense system [16]. This preventive effect of AOX on free radicals could be explained by its components, in this study, AOX contains primary (Ethoxyquin, Tertiary butyl hydroquinone) and secondary antioxidant (Citric acid). The primary antioxidants, as free radical scavengers, can inactivate free radicals during interaction with peroxy radical and in the termination reaction with another peroxy radical [15]. Citric acid, as a multiple carboxylic acid compounds, acted as chelators for prooxidant or catalyst metal ions providing H to primary antioxidants, thus decomposed hydroperoxide to nonradical species [15].

**Table 5:** Effects of oxidized oil with or without antioxidant (AOX) on oxidative status of sows

	Oil quality		AOX		SEM	*P* value						
	FO	OO	FO + AOX	OO + AOX		AOX	Oil	Day	AOX × Oil	Day × AOX	Day × Oil	Day × AOX × Oil
VE												
Overall Mean	6.46	6.05	6.76	6.64	0.24	0.18	0.53	0.00	0.63	0.42	0.63	0.91
D 85 of gestation	8.34	8.46	8.21	8.30	0.42							
D 1 of lactation	4.89	4.46	5.29	5.11	0.39							
D 21 of lactation	6.66	6.15	7.01	6.98	0.42							
VC												
Overall Mean	1.18	1.23	1.10	1.23	0.09	0.92	0.82	0.00	0.62	0.99	0.86	0.97
D 85 of gestation	2.40	2.45	2.33	2.47	0.17							
D 1 of lactation	0.59	0.50	0.53	0.58	0.18							
D 21 of lactation	0.74	0.71	0.70	0.71	0.18							
Total SOD												
Overall Mean	67.07	62.44	66.80	63.45	2.50	0.81	0.03	0.00	0.88	0.9	0.4	0.85
D 85 of gestation	60.81	59.03	61.20	60.23	2.87							
D 1 of lactation	82.17^a^	73.97^b^	81.89^a^	76.43^ab^	2.87							
D 21 of lactation	56.96	55.34	57.31	53.68	3.06							
CuZn-SOD												
Overall Mean	32.86	31.59	31.51	31.95	1.15	0.86	0.95	0.00	0.54	0.88	0.92	0.85
D 85 of gestation	31.34	30.22	29.96	29.88	2.01							
D 1 of lactation	41.57	40.19	40.53	42.33	1.88							
D 21 of lactation	24.41	24.93	24.05	24.67	2.00							
Mn-SOD												
Overall Mean	37.45	33.26	36.27	35.08	1.26	0.33	0.05	0.00	0.22	0.5	0.53	0.49
D 85 of gestation	31.74	31.62	32.23	31.34	2.13							
D 1 of lactation	44.49	38.01	44.46	44.74	2.1							
D 21 of lactation	34.74	29.09	33.27	31.13	2.13							
GPx												
Overall Mean	1291	1239.6	1287.52	1278.05	52.35	0.57	0.25	0.00	0.85	0.78	0.63	0.99
D 85 of gestation	1671	1634.2	1661.53	1632.64	85.55							
D 1 of lactation	869	837.65	871,095	865.94	92.5							
D 21 of lactation	1278	1132.1	1345.69	1225.34	88.4							
MDA												
Overall Mean	1.14	1.28	1.12	1.14	0.04	0.08	0.06	0.02	0.22	0.3	0.1	0.54
D 85 of gestation	1.13	1.09	1.15	1.1	0.07							
D 1 of lactation	1.13	1.27	1.03	1.1	0.07							
D 21 of lactation	1.16b	1.47a	1.18b	1.24b	0.07							

Means in rows with different superscripts markedly differ, *P* < 0.05. *n* = 8

Treatments: FO means fresh oil; OO means oxidized oil; FO + AOX means fresh oil plus AOX; OO + AOX means oxidized oil plus AOX

*VE* vitamin E, *VC* vitamin C, *Total SOD* total superoxide dismutase, *CuZn-SOD* copper and zinc-containing superoxide dismutase, *Mn-SOD* manganese-containing superoxide dismutase, *GPx* glutathione peroxidase, *MDA* malonaldehyde

**Table 6:** Effects of oxidized oil with or without antioxidant (AOX) on placental gene expression of antioxidant enzymes in sows

	Oil quality		AOX		SEM	*P* value		
	FO	OO	FO + AOX	OO + AOX		AOX	Oil	AOX × Oil
GPX	1	2.44	1.66	1.09	0.23	0.041	0.014	0.007
NOS	1	4.81	2.26	1.04	0.01	0.121	0.091	0.065
SOD	1	2.5	1.09	0.92	0.69	0.023	0.006	0.014
CAT	1	2.71	1.54	2.13	0.01	0.083	0.013	0.159

Treatments: FO means fresh oil; OO means oxidized oil; FO + AOX means fresh oil plus AOX; OO + AOX means oxidized oil plus AOX. *n* = 8

*GPx* glutathione peroxidase, *NOS* nitric oxide synthase, *SOD* superoxide dismutase, *CAT* catalase

Sow placenta provides extensive and intimate interface between maternal and fetal blood streams to exchange nutrients and wastes [32]. It was demonstrated that oxidative stress impaired placental development and function by affecting the placental AKT-mTOR signal pathway [33]. As a rapidly developing organ in late gestation [13], sow placenta may be sensitive for the systematic oxidative status. By analyzing the placental gene expression of antioxidant enzymes (Table 6), strikingly, placental gene expression of GPX and SOD were significantly higher in sows fed oxidized oil (approximately 2.5 fold increase, *P* < 0.05) relative to sows fed fresh oil, whereas their gene expressions were consistently normalized by supplementing AOX (*P* < 0.05). The interaction of oil quality and AOX was detected for gene expression of GPX and SOD. It is possible that the antioxidant system of sow placenta had an adaptive response to oxidative stress, as similar results by Lappas et al., who demonstrated that placental antioxidant system has a proper capability to compensate for the oxidative stress through increasing gene expression of antioxidant enzymes [34].

## Conclusion

In summary, feeding oxidized oil did not markedly affect reproductive performance in addition to the decreased feed intake during lactation. Milk composition and oxidative status may be deteriorated in sows fed oxidized oil but partially improved by dietary AOX supplementation.

## Abbreviations

AOX: Antioxidant
SOD: Superoxide dismutase
MDA: Methane dicarboxylic aldehyde
GPX: Glutathione peroxidase
ROS: Reactive oxygen species
FO: Fresh corn oil
OO: Oxidized corn oil
FO + AOX: FO plus 200 mg/kg AOX
OO + AOX: OO plus 200 mg/kg AOX
CAT: Catalase
NOS: nitric oxide synthase
